# Translating recent results from the Cardiovascular Outcomes Trials into clinical practice: recommendations from the Central and Eastern European Diabetes Expert Group (CEEDEG)

**DOI:** 10.1186/s12933-017-0622-7

**Published:** 2017-10-23

**Authors:** Guntram Schernthaner, Roger Lehmann, Martin Prázný, Leszek Czupryniak, Kristine Ducena, Peter Fasching, Andrej Janež, Avraham Karasik, Peter Kempler, Emil Martinka, Marina V. Shestakova, Lea Smirčić Duvnjak, Tsvetalina Tankova

**Affiliations:** 1 Department of Medicine I, Rudolfstitung Hospital, Vienna, Austria; 2 Division of Endocrinology and Diabetes of the University Hospital, Zurich, Switzerland; 3 Diabetes Centre, Charles University and General Faculty Hospital, Prague, Czech Republic; 4 Department of Diabetology and Internal Medicine, Warsaw Medical University, Warsaw, Poland; 5 Faculty of Internal Medicine, University of Latvia, Riga, Latvia; 6 5th Medical Department, Wilhelminenspital, Vienna, Austria; 7 Department of Endocrinology, Diabetes and Metabolic Diseases, University Medical Centre, Ljubljana, Slovenia; 8 Chaim Sheba Medical Centre, Tel Hashomer, Israel; 9 Department of Medicine, Semmelweis University, Budapest, Hungary; 10 National Institute of Endocrinology and Diabetology, Lubochna, Slovakia; 11 Endocrinology Research Centre, Moscow, Russia; 12 Department of Endocrinology and Metabolic Diseases, Vuk Vrhovac University Clinic for Diabetes, Endocrinology and Metabolic Diseases, Merkur University Hospital, School of Medicine, University of Zagreb, Zagreb, Croatia; 13 Clinical Centre of Endocrinology, Medical University, Sofia, Bulgaria

**Keywords:** Type 2 diabetes, Anti-glycaemic drugs, Cardiovascular disease, Renal disease

## Abstract

**Aims:**

These recommendations aim to improve care for patients with type 2 diabetes (T2D) at high cardiovascular (CV) risk in Central and Eastern Europe. Cardiovascular disease (CVD) and/or chronic kidney disease (CKD) are major interdependent comorbidities in patients with T2D, accounting for 50% of mortality. Following recent CV outcomes trial (CVOT) results, including those from EMPA-REG OUTCOME^®^, LEADER^®^, SUSTAIN™-6 and, most recently, the CANVAS study, it is essential to develop regional expert consensus recommendations to aid physicians in interpreting these newest data to clinical practice.

**Methods:**

The Central and Eastern European Diabetes Expert Group (CEEDEG) followed a Delphi method to develop treatment algorithms to aid physicians in the clinical management of patients with T2D at high CV risk.

**Results:**

In light of the latest CVOT results, and in particular the EMPA-REG OUTCOME^®^ and LEADER^®^ trials, the diagnosis, assessment, treatment choice and monitoring of patients with T2D and established CVD and/or CKD have been considered together with existing guidelines and presented in two reference algorithms. In addition, adherence, special prescribing considerations and a proposed multidisciplinary management approach have been discussed and are presented with the proposed algorithms.

**Conclusions:**

The latest available high-level evidence on glucose-lowering drugs has enabled CEEDEG to develop practical consensus recommendations for patients with established CVD and/or CKD. These recommendations represent an update to international and country-level guidelines used for these patients, with the aim of providing a resource not only to endocrinologists, but to cardiologists, nephrologists and primary care physicians in the region.

**Electronic supplementary material:**

The online version of this article (doi:10.1186/s12933-017-0622-7) contains supplementary material, which is available to authorized users.

## Background

For patients with type 2 diabetes (T2D), cardiovascular disease (CVD) is the single most common cause of mortality [1]. The average life expectancy of a 60-year-old male with T2D and no history of cardiovascular (CV) disease (CVD) is 12 years less than his counterpart without diabetes, mostly owing to a 58% increase in risk of CV death [2].

Historical studies investigating the potential benefits of intensive glucose-lowering therapy have shown mixed results [3, 4], and concerns were raised that some anti-diabetic agents might even be increasing CV risk in patients with T2D; a particular concern in light of the high prevalence of CV and/or renal co-morbidities in this patient population. Meta-analyses revealed that rosiglitazone was associated with a significant increase in the risk of myocardial infarction (MI) compared with patients not receiving rosiglitazone, and an increase in the risk of CV death that had borderline significance [5, 6]. The later US veterans affairs diabetes trial showed, however, that rosiglitazone use in older patients with T2D was associated with decreased risk of the primary CV composite outcome and CV death, and that rosiglitazone use did not lead to a higher risk of MI [7]. Meta-analyses across study results are not designed to assess superiority, are not prospective and are likely to necessitate the inclusion of heterogeneous study designs. To address this safety concern, the US Food and Drug Administration (FDA) and the European Medicines Agency (EMA) have therefore asked for proof of CV safety, which includes CV outcomes trials (CVOTs) being initiated on all new anti-diabetic agents to provide prospective, statistically powered assessments in patients with T2D in order to rule out excess CV risk [8–10].

CVOTs for several anti-diabetic drugs have been published in the past few years. Although these new anti-diabetic agents generated mostly neutral CV outcomes, providing welcome evidence that most of these drugs do not increase CV risk for patients with T2D, there were mixed results overall, as shown in Table 1 [4, 10].

**Table 1:** Key cardiovascular and renal outcomes for CVOTs of glucose-lowering agents

	Reduction in 3-point MACE	Reduction in CV death	Reduction in all-cause mortality	Reduction in hospitalisation for HF	Reduction in doubling of serum creatinine
EMPA-REG OUTCOME^®^					
RRR (%)	14	38	32	35	44
*p* value	0.04	< 0.001	< 0.001	0.002	< 0.001
LEADER^®^					
RRR (%)	13	22	15	13	12
*p* value	0.01	0.007	0.02	NS	NS
SUSTAIN™-6					
RRR (%)	26	2	+ 5	+ 11	+ 28
*p* value	0.02	NS	NS	NS	NS

This is not a head-to-head comparison

SUSTAIN-6™ was a non-inferiority study, and testing for superiority was not a pre-specified endpoint [22, 23, 25, 28, 29, 33]

*CVOT* cardiovascular outcomes trial, *MACE* major adverse cardiovascular events (cardiovascular death, non-fatal myocardial infarction or non-fatal stroke), *RRR* relative risk reduction

In light of these mixed results, the swift incorporation of CVOT findings into international and country-level guidelines is therefore necessary to facilitate treatment decisions for improved patient outcomes. Many guidelines for the treatment of T2D require further updates, including, importantly, the Joint Position Statement of the European Association for the Study of Diabetes (EASD)/American Diabetes Association (ADA) and the American College of Physicians (ACP) [11, 12]. The guidelines of the European Society of Cardiology (ESC) and the ADA, however, have recently incorporated recommendations based on the CVOT results [13, 14]. The Central and Eastern European Diabetes Expert Group (CEEDEG) was created as a responsive team of experts, providing regular and up-to-date clinical recommendations to be used as a companion resource for the region, together with the EASD/ADA position statement. By providing such regular follow-up publications, it is anticipated that important data on safety and both positive and negative outcomes can be rapidly disseminated. CEEDEG is composed of 14 members, six of whom have been involved in the generation of their national clinical guidelines, including the Swiss Society of Endocrinology and Diabetes (SSED/SGED) [15], the Hungarian Diabetology Society [16], the Slovak Diabetes Association [17], the Russian Association of Endocrinologists [18], and the Austrian Society for Diabetes (ÖDG) [15, 19]. All members hold senior positions in medical schools in their respective countries and belong to, or serve on, a variety of professional bodies throughout Central and Eastern Europe. The recommendations herein have been derived via a Delphi process, which is a structured tool to achieve unbiased consensus [20].

## Methods

CEEDEG was formed to provide a panel of experts in Central and Eastern Europe who could regularly review, interpret and translate new data from clinical studies into clinical recommendations for everyday practice.

The process was structured to generate expert clinical recommendations to complement the current EASD/ADA guidelines in light of the data from EMPA-REG OUTCOME^®^, LEADER^®^, SUSTAIN™-6 and the other CVOTs that had been published at the time of the Delphi process [21–25]. Agreeing on the necessity of producing such recommendations was an inclusive initial part of the proceedings.

A semi-structured questionnaire with multi-factor open questions was circulated (Additional file 1). The questionnaire related to patients with T2D and established cardiac and/or renal disease, as these are the patient populations who would most benefit from recommendations based on CVOT data. The recommendations generated were sorted, de-duplicated and used to create a second questionnaire (Additional file 2), in which respondents scored each proposed recommendation on a Likert-like scale (1-totally disagree; 2-disagree; 3-unsure; 4-agree; 5-totally agree). Only those recommendations for which at least 80% of respondents awarded a score of 4 or 5 were considered to have reached consensus. Respondents were encouraged to add additional comments, suggestions or refinements to the proposed statements. Additional suggestions and refinements were included in a third-round questionnaire, which was scored in the same way as the second (Additional file 3).

Following the Delphi process, a face-to-face meeting of CEEDEG was convened with the aim of reaching final consensus and addressing any outstanding issues from the process. In-depth discussions were held and grouped according to clinical expertise to refine and clarify points of particular complexity, then all members met to verify or reject statements together, thereby achieving a full and final consensus.

## Recommendations of CEEDEG

**Fig. 1:**
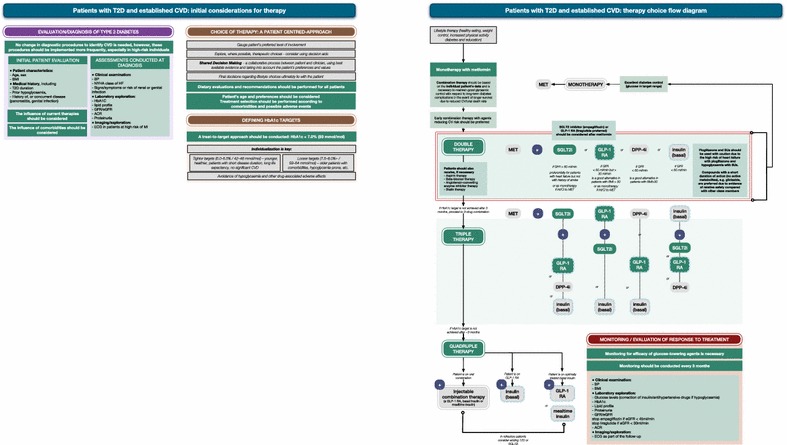
Treatment algorithm for patients with type 2 diabetes (T2D) and established cardiovascular disease (CVD): initial considerations for therapy and therapy choice flow diagram. Items in green signify the items of specific focus in these recommendations. Dashed lines indicate injectable therapies. *ACR* albumin:creatinine ratio, *BMI* body mass index, *BP* blood pressure, *CKD* chronic kidney disease, *CVD* cardiovascular disease, *DPP-4i* dipeptidyl peptidase 4 inhibitor, *ECG* electrocardiogram, *GFR* glomerular filtration rate, *eGFR* estimated *GFR*, *DPP-4-i* DPP-4 inhibitor, *GLP-1 RA* glucagon-like peptide 1 receptor agonist, *HF* heart failure, *hypo* hypoglycaemia, *int/CI* intolerance or contraindication, *MET* metformin, *NYHA* New York Heart Association, *SGLT2i* sodium–glucose co-transporter 2 inhibitor, *SU* sulfonylurea, *T2D* type 2 diabetes, *TZD* thiazolidinedione

**Fig. 2:**
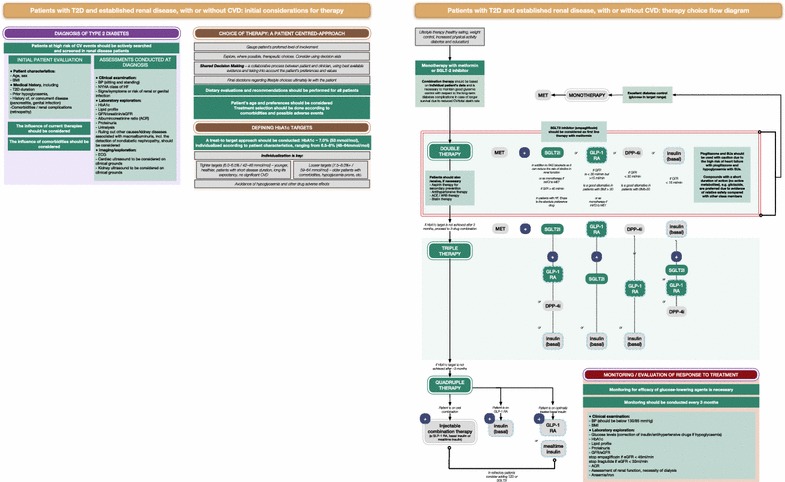
Treatment algorithm for patients with type 2 diabetes (T2D) and established chronic kidney disease (CKD), with or without cardiovascular disease (CVD): initial considerations for therapy and therapy choice flow diagram. Items in green signify the items of specific focus in these recommendations. Dashed lines indicate injectable therapies. *ACR* albumin:creatinine ratio, *BMI* body mass index, *BP* blood pressure, *CKD* chronic kidney disease, *CVD* cardiovascular disease, *DPP-4i* dipeptidyl peptidase 4 inhibitor, *ECG* electrocardiogram, *GFR* glomerular filtration rate, *eGFR* estimated GFR, *DPP-4-i* DPP-4 inhibitor, *GLP-1 RA* glucagon-like peptide 1 receptor agonist, *HF* heart failure, *hypo* hypoglycaemia, *int/CI* intolerance or contraindication, *MET* metformin, *NYHA* New York Heart Association, *SGLT2i* sodium–glucose co-transporter 2 inhibitor, *SU* sulfonylurea, *T2D* type 2 diabetes, *TZD* thiazolidinedione

CEEDEG noted that for those patients who have T2D but do not have established CVD or chronic kidney disease (CKD), the current EASD/ADA guidelines [11] should be followed as no further data on therapies for these patients have been published. However, for patients with either or both of these comorbidities, two algorithms were developed: one for patients with T2D and established CVD (Fig. 1), and the other for patients with T2D and established CKD with or without established CVD (Fig. 2). It is hoped that these additional tools will be used alongside the European guidelines as an aid to decision-making when prescribing glucose-lowering therapies. It should be emphasised that all agents referred to within these recommendations should be used in accordance with the relevant summary of product characteristics or prescribing information for the country in which they are being prescribed.

### Diagnosis/assessment

The evaluation of all patients should include the following parameters: age, sex, height, weight and body mass index (BMI). The medical history should document the time since diagnosis of T2D, any episodes of prior severe hypoglycaemia and the presence of, or history of, concurrent pancreatitis or genital infection.

For patients with established CKD, any retinopathy, autonomic neuropathy [26], haematological disorders or renal infections should also be ascertained. Patients with established CKD, and at high risk of CV events but with as yet undiagnosed CVD, should be actively screened for the presence of CV risk factors.

The presence of any other comorbidities and current pharmacological therapies should be determined and taken into consideration when making treatment decisions, to ensure that the patient receives optimally individualised care to achieve the best possible outcome.

A comprehensive investigative work-up is needed to make best-practice treatment decisions. Clinical, laboratory, and, for certain patients, other physiological evaluations should be performed:

- Clinical: blood pressure sitting and standing, and classification of heart failure (HF) according to New York Heart Association functional criteria [27].
- Laboratory:
  - HbA1c levels; lipid profile; glomerular filtration rate (GFR)/estimated GFR (eGFR), urinary albumin/creatinine ratio (UACR) and proteinuria.
  - For patients with CKD, urinalysis.
  - Brain natriuretic peptide (BNP)/N-terminal prohormone of BNP (NT-proBNP) should be considered in patients having symptoms or signs of HF.
  - Other causes/kidney diseases associated with macroalbuminuria, including the detection of nondiabetic nephropathy, should be ruled out.
  - Physiological measurements:
    - For patients who are at particular risk of MI, electrocardiogram (ECG) should be performed.
    - For patients with CKD, kidney/cardiac ultrasound should be considered depending on clinical need.

### Clinical management

CEEDEG, in common with the EASD/ADA guidelines, recommended a patient-centric approach to treatment to enable the optimal combination of ideal therapy and patient compliance.

### Adherence rates and patient education

Several factors need to be taken into consideration to build on long-term adherence rates and good self-management by patients:

- Therapeutic choices should be explored with the patient, making use of decision aids where relevant, and should take place within the context of the patient’s priorities, goals and preferred level of involvement.
- Treatment strategies should be kept as simple as possible to reduce the apparent treatment burden, e.g. the use of dual-agent therapies.
- Treatments should be carefully and thoroughly explained to patients on multiple occasions, and reiterated by all practitioners involved in the patient’s care.
- The patient’s age should also be considered when selecting treatment, taking into account age-related comorbidities and the special repercussions of possible adverse events, such as falls in elderly or frail patients.
- Full dietary evaluation (rather than simple caloric intake) should be performed for all patients.
- The agreed management program should then be implemented and run by clinical specialists and primary care physicians, ideally involving a dietitian and, if needed, a psychologist (Table 2).

**Table 2:** Specialities for inter-professional, multidisciplinary team-based type 2 diabetes care for a comprehensive multifactorial risk-reduction strategy in the context of cardiovascular comorbidity

Speciality	Key functions and areas of responsibility
Diabetologist/endocrinologist	Therapy induction including patient education: explanation of therapy choice, side-effects, complications, acute emergencies Sets BP, lipid, HbA1c targets Trained in cardiology/nephrology/internal medicine
Internist	Follow-up care, including monitoring of BP, lipid targets and controls, ECG, echocardiagram imaging Trained in diabetology (patients with diabetes who are complicated to treat should always be referred to diabetologists/endocrinologists)
Nephrologist	Renal function, ACR, BP control Lipid targets
Cardiologist	Essential for CVD and CV risk management Trained in diabetology
Nutritionist/dietician	Dietary counselling at therapy induction A key figure in patient education together with diabetologist/endocrinologist Monitoring of glucose control and intake, and body weight Individual diet plans for weight reduction if needed, and/or long-term weight maintenance
Nurses	Education programmes Individual support BMI monitoring
Certified diabetes educator	Smoking cessation Diabetes education at treatment induction and for re-enforcement Education programmes, team work Consistent and structured education that is crucial for adherence, motivation and minimising barriers to treatment

*ACR* albumin:creatinine ratio, *BMI* body mass index, *BP* blood pressure, *CV* cardiovascular, *CVD* CV disease, *ECG* electrocardiogram, *ECHO* echocardiogram

### Glycaemic control

CEEDEG recommended a treat-to-target approach based on reducing HbA1c levels to below 7.0% (53 mmol/mol); however, individualisation is key [28]. For elderly and frail patients with significant CVD or CKD co-morbidities, or who are prone to hypoglycaemia, more relaxed targets in the range of 7.5 − 8.0 + % (58.5–63.9 mmol/mol) may be applied. This can be tightened to 6.0–6.5% (42.1–47.5 mmol/mol) in younger, healthier patients or those without significant CVD. Overall, it is important to set the target HbA1c level appropriately for each individual to avoid hypoglycaemia and any other drug-related adverse effects.

### Cardiovascular protection

As discussed in the introduction, CV mortality remains the main cause of death in this patient group despite recent advances in therapies for T2D. Intensive glycaemic control does not always lead to better CV outcomes [29]; therefore, focusing solely on glucose lowering may not help to address macrovascular complications in T2D. Although the target of modern diabetes therapies in the recent past has been a safe and effective blood glucose reduction [11], recent results of trials dedicated to CV outcomes clearly demonstrate that innovative diabetic drugs such as empagliflozin and liraglutide can add significant benefit to outcomes for patients with T2D in terms of a reduction in CV death [23, 30–32]. These important clinical results have led to a new duality paradigm for T2D treatment, whereby both the improvement of glycaemic control and the reduction of CV morbidity and mortality become an integral part of the treatment of T2D. Therefore, CV risk and protection should always be considered when determining therapeutic regimens for patients with T2D.

### Type 2 diabetes therapeutic regimens

#### Monotherapy

Although monotherapy with metformin is commonly used in patients with no CV comorbidities, the early combining of metformin therapy together with agents that reduce CV risk should be preferred in patients with T2D and established CVD.

Progressing patients from mono- to combination therapy should be considered on an individual basis, depending on the patient’s investigation data, and is recommended to achieve good glycaemic control and better CV and renoprotective outcomes.

#### Combination therapy

Current glucose-lowering therapies comprise six classes, each with differing combinations of attributes over and above decreasing HbA1c, such as effects on CV risk, renal outcomes, blood pressure and weight. Suitability for use in the presence of nephropathy should be considered when choosing agents for multiple combinations (Table 3).

**Table 3:** Current glucose-lowering therapies and their key attributes. Adapted from the SGED/SSED guidelines [13, 15], except where indicated

Class	Beneficial effect on CV outcomes	Beneficial effect on HbA1c levels	Effect on weight	Hypoglycaemic risk
*Metformin* (oral)	Moderate (long-term)	Moderate	Moderate decrease	Neutral
SGLT2 inhibitors (oral)				
*Empagliflozin* ^e^	High (empagliflozin and canagliflozin)	Moderate to high	High decrease	Neutral
Canagliflozin				
Dapagliflozin				
GLP-1 receptor agonists (injection)				
*Liraglutide*	High (liraglutide and semaglutide)	High	Very high decrease	Neutral
Semaglutide				
Exenatide				
Exenatide LAR				
Dulaglutide^a^				
DPP-4 inhibitors (oral)				
*Sitagliptin* ^e^	Neutral	Moderate	Neutral	Neutral
Alogliptin^e^				
Linagliptin^e^				
Saxagliptin				
Vildagliptin^e^				
Insulins (injection)				
*Insulin degludec*	Neutral	High	Neutral	High
*Insulin glargine*				
TZDs (oral)				
Pioglitazone	High?^b^	Moderate?^c^	Increase^d^	
SUs (oral)				
*Gliclazide*	Neutral	Moderate	Neutral	Moderate
Glimepiride				

Agents in italics have a higher level of evidence for reduction of micro- and macrovascular complications and mortality or fewer side-effects

*CV* cardiovascular, *DPP-4* dipeptidyl peptidase 4, *GLP-1* glucagon-like peptide 1, *LAR* long-acting release, *SGLT2* sodium–glucose co-transporter 2, *SU* sulfonylurea, TZD thiazolidinedione

^a^A meta-analysis of the CV safety of dulaglutide in patients with T2D [52]

^b^Astudy that predates the CVOT mandate demonstrated significant reduction in a CV composite outcome. The study was a post hoc analysis of a prospective clinical trial [41, 53]

^c^A double-blind, randomised trial that compared pioglitazone with metformin as monotherapies found that the HbA1c reduction was similar between the two drugs [42, 54]

^d^An observational prescription-event monitoring study that showed treatment with pioglitazone was associated with a low incidence of hypoglycaemia [43, 55]

^**e**^Also available in combination with metformin

### Dual therapy

The choice of agent for initial combination with metformin can be streamlined on the basis of patient comorbidities and characteristics.

### SGLT2 inhibitors

For patients with T2D and CVD, sodium–glucose co-transporter 2 (SGLT2) inhibitors should be considered for use after metformin monotherapy in those with a GFR > 60 ml/min (or > 45 ml/min or above in countries where this is the indicated threshold for empagliflozin/canagliflozin prescribing).

Current evidence from the EMPA-REG OUTCOME^®^ trial showed that empagliflozin significantly reduced the time to the first occurrence of the primary composite endpoint of 3-point major adverse cardiovascular events (3-P MACE): CV death, non-fatal MI or non-fatal stroke (HR: 0.86; 95% CI 0.74–0.99) [21, 30, 33, 34]. The primary endpoint improvement was mostly driven by a 38% relative risk reduction (RRR) in the key outcome of CV mortality (HR 0.62; 95% CI 0.49–0.77). Hospitalisation for HF, a defined secondary outcome, was also significantly lower in the empagliflozin group compared with placebo (HR 0.65; 95% CI 0.50–0.85; *p* < 0.002), likewise, there was a significant lowering of all-cause mortality with the addition of empagliflozin to standard of care (HR 0.68, 95% CI 0.57–0.82; *p* < 0.001). This SGLT2 inhibitor should therefore be preferentially chosen for patients at high risk of CV events [33].

For patients with T2D and CKD, empagliflozin was recommended in early combination with metformin and renin–angiotensin system (RAS) blockade, as it can reduce the rate of renal decline. Incidence of or worsening nephropathy occurred in fewer patients in the empagliflozin group than in the placebo group (HR 0.61; 95% CI 0.53–0.70). In patients treated with empagliflozin compared with placebo there was a RRR of the doubling of serum creatinine levels of 44%, and a 55% RRR of renal-replacement therapy being initiated [30].

Owing to the documented CV benefits mentioned above, empagliflozin should be considered as the drug of preference for patients with T2D and CKD who also have established CVD.

Empagliflozin may also be used as a monotherapy if the patient is intolerant to metformin or if metformin is contraindicated.

### GLP-1 receptor agonists

Glucagon-like peptide 1 (GLP-1) receptor agonists (preferentially liraglutide) should be considered in patients with a GFR below 60 ml/min but greater than 30 ml/min. The results of the LEADER^®^ CVOT of liraglutide in patients with T2D and at high risk of CV events showed lower CV-related and all-cause mortalities in patients treated with liraglutide compared with placebo (HR 0.78; 95% CI 0.66–0.93 and HR 0.85; 95% CI 0.74–0.97, respectively) [23]. These agents are also recommended for patients with a BMI > 30, owing to their documented benefits in weight reduction. GLP-1 receptor agonists may also be used as a single agent in patients intolerant to metformin.

### DPP-4 inhibitors

CVOTs of the dipeptidyl peptidase 4 (DPP-4) inhibitors saxagliptin, alogliptin and sitagliptin have shown neutral results in which neither an increase nor a decrease was seen in the rate of ischaemic events in patients treated with these agents when compared with placebo, although the rate of hospitalisation for HF was increased for saxagliptin [35–37]. For patients who are unsuitable for either an SGLT2 inhibitor or a GLP-1 receptor agonist, such as those with an eGFR below 60 ml/min (30 ml/min for patients with CKD), DPP-4 inhibitors (oral) may be considered.

### Insulin

Basal insulin injections are an option for all patients However, it is recommended that their early use is restricted to those patients with a GFR below 60 ml/min (15 ml/min for patients with CKD) and who cannot be prescribed any of the aforementioned agents. Otherwise, this treatment option should be reserved for later use.

### Other type 2 diabetes treatment agents

Pioglitazone and sulfonylureas (SUs) should be used with caution in all patients, owing to the high risk of congestive HF in the former and hypoglycaemia in the latter of these agents. Where prescribing these classes is necessary, e.g. for financial/reimbursement reasons, compounds with a short duration of action (no active metabolites), such as gliclazide, are preferred, owing to the evidence of relative safety compared with other class members.

### Triple therapy

If, after 3 months of dual combination therapy, the HbA1c target is not achieved, the addition of a third therapeutic agent should be considered as second-line combination therapy. Here, the choice of additional agent should largely depend on responses to the existing combination, as well as on the patient’s co-morbidities.

For all patients who are already taking metformin plus an SGLT2 inhibitor for its CV risk-reduction benefits, the recommended third agents are, in order of preference, a GLP-1 receptor agonist, a DPP-4 inhibitor or basal insulin. CEEDEG recommended that patients who do not show a decrease in HbA1c level from treatment with empagliflozin should not be simply switched to a GLP-1 receptor agonist, owing to the documented important cardio- and renoprotective effects of empagliflozin.

The agents that are of most benefit to patients with T2D and CVD who are already being treated with metformin and a GLP-1 receptor agonist are either an SGLT2 inhibitor or basal insulin.

For those patients who received a DPP-4 inhibitor with metformin in the first instance, then either the DPP-4 inhibitor should be replaced by a long-acting GLP-1 receptor agonist, or a basal insulin or gliclazide should be prescribed. For patients who received basal insulin with metformin, add an SGLT2 inhibitor, a GLP-1 receptor agonist or a DPP-4 inhibitor, depending on the GFR.

A quadruple combination should be initiated if, after a further 3 months of triple combination therapy, the patient has still not achieved their individual target HbA1c level. For those patients who are taking oral therapies, an injectable therapy should be added: a GLP-1 receptor agonist, basal insulin or mealtime insulin. If the patient is receiving a GLP-1 receptor agonist, then basal insulin should be the next agent. If the patient is already on optimally treated basal insulin, then either a GLP-1 receptor agonist or mealtime insulin should be added, depending on whether they are already taking a GLP-1 receptor agonist. If patients remain refractory to treatment, then consider adding a thiazolidinedione (TZD) or an SGLT2 inhibitor, also depending on the existing therapeutic agents that the patient is receiving.

#### Monitoring/evaluation of response to treatment

As suggested above, to determine the efficacy of glucose-lowering treatments, and to monitor changes in CV risk factors, regular monitoring of the patient should be conducted at 3-monthly intervals. The parameters that should be evaluated are similar to those for initial assessment, and should remain comprehensive to manage both glucose levels and CV risk.

Clinical examination should include blood pressure and BMI. The laboratory tests should ascertain glucose levels to allow for correction of insulin/anti-hypertensive drugs if the patient has been experiencing hypoglycaemia. Laboratory tests should also measure HbA1c level, lipid profile, and proteinuria, GFR/eGFR and UACR.

The SGLT2 inhibitor empagliflozin should be withdrawn if the eGFR has reduced to below 45 ml/min as indicated; however, data from clinical studies show that benefits may still be seen in patients down to an eGFR of 30 ml/min [30, 33]. The GLP-1 receptor agonist liraglutide should also be withdrawn when a minimum eGFR of 30 ml/min is reached.

A repeat ECG should be performed as part of the follow-up to monitor any changes in CV status.

#### Cardiac/lipid control

In addition to glucose-lowering agents, patients should also receive an anti-platelet aggregation agent, beta-blocker, angiotensin-converting enzyme (ACE) inhibitor, angiotensin receptor blocker (ARB) and statin therapies if indicated.

#### Special prescribing considerations

Although basal and other insulins have been somewhat superseded by the new generation of anti-hyperglycaemic drugs, they should not be excluded where needed. When prescribing insulins, however, the latest evidence should be considered, which suggests that insulin is best used alongside more modern drugs. Insulin has good glucose-lowering effects, but can lead to hypoglycaemia if not carefully prescribed according to the patient’s lifestyle; therefore, its therapeutic effect should be carefully evaluated. For patients who are receiving other glucose-lowering agents that may offer other benefits, such as CV and renal protection, but who are not achieving their target HbA1c levels, insulin in combination with more modern drugs may enable them to control both aspects of their condition. CEEDEG felt that the evidence for the use of insulin as a monotherapy does not warrant its use in this way, especially for the older, short-acting forms.

#### Elderly patients

Patients in the upper age ranges (75 years and older) are highly variable in terms of individual fitness and general lifestyles. Although care should be taken to avoid hypoglycaemia and its associated increased risk of falls when treating frail elderly patients with T2D and CVD, it should be taken into account that the maximum CV benefits derived from the SGLT2 inhibitor empagliflozin in the EMPA-REG OUTCOME^®^ study were seen among the older participants, and this population is also more likely to suffer from CV and renal comorbidities. Preventative measures should therefore be taken to avoid hypovolaemia when prescribing empagliflozin in this patient group. In the LEADER^®^ study of liraglutide, however, patients aged over 60 years experienced fewer CV events in the placebo arm than in the treatment arm [22, 23].

As with all other therapies, therefore, patient preference should be taken together with the medical history when choosing an appropriate agent or agents.

#### Class effect

No strong evidence has yet been found for a class effect regarding positive CV outcomes of anti-diabetic agents (Table 4 and Additional file 4: Table S1), although overall, no increased CV risk has been observed [10]. For example, CVOTs for GLP-1 receptor agonists and DPP-4 inhibitors have shown mixed results depending on the individual drug [23, 25, 35–38]. In the SGLT2 inhibitor class, although the EMPA–REG OUTCOME^®^ study and CANVAS program differ (for example by study design and the pharmacological characteristics of the drugs), the results of both trials showed a significant reduction on the composite endpoint (3P-MACE); however, individual components of 3P-MACE and key secondary outcomes differed greatly [33, 39]. In the EMPA-REG OUTCOME^®^ study, CV death had a relative risk reduction of 38% (95% CI 0.49–0.77), whereas in the CANVAS Program CV death was not significantly reduced (13% RRR, 95% CI 0.72–1.06). Additionally, in the EMPA-REG OUTCOME^®^ study the key secondary outcomes of all-cause mortality (RRR 32%, 95% CI 0.57–0.82, p < 0.001), HHF (RRR 35%, 95% CI 0.50–0.85, p < 0.001) and the composite renal outcome (RRR 46%, 95% CI 0.31–0.85, p < 0.001) all showed a statistically significant benefit when adding empagliflozin versus placebo to standard of care [33]. By contrast, in the CANVAS Program none of the secondary outcomes showed a statistical benefit [39]. This is in part owing to all-cause mortality not being significantly reduced in the CANVAS Program (13% RRR, 95% CI 0.74–1.01) after which all other secondary outcomes analysed in the sequential hypothesis testing plan can only be interpreted as exploratory, such as HHF (33% RRR, 95% CI 0.52–0.87) and the composite renal outcome (RRR 40%, 95%CI 0.47–0.77). In contrast, EMPA-REG OUTCOME^®^ was powered sufficiently to achieve significance in both HHF (RRR 35%, 95% CI 0.5–0.85, p < 0.001) and the composite renal outcome (RRR 46%, 5% CI 0.31–0.85, p < 0.001) [30, 33, 39]. An additional difference between SGLT2 inhibitors is the observation that there was an increased signal for lower limb amputations related to canagliflozin use in the CANVAS studies, an outcome that, although observed at a higher rate in patients with diabetes than in the general population, has not been observed in any of the empagliflozin or dapagliflozin studies to date [39–44].

**Table 4:** Table of 3-point MACE for CVOT studies to date. Adapted from [39, 50, 51]

	Antidiabetic drug	3P-MACE hazard ratio (HR)	*p* value
PROactive	Pioglitazone	0.84 (95% CI 0.72–0.98)	0.02
ORIGIN	Insulin glargine	1.02 (95% CI 0.94–1.11)	NS
SAVOR	Saxagliptin	1.00 (95% CI 0.89–1.12)	NS
EXAMINE	Alogliptin	0.96 (95% CI 0.80–1.15)	NS
ELIXA	Lixisenatide	1.02 (95% CI 0.89–1.17)	NS
TECOS	Sitagliptin	0.98 (95% CI 0.89–1.08)	NS
EMPA-REG OUTCOME^®^	Empagliflozin	0.86 (95% CI 0.74–0.99)	0.038
LEADER^®^	Liraglutide	0.87 (95% CI 0.78–0.97)	0.01
SUSTAIN™-6	Semaglutide	0.74 (95% CI 0.58–0.95)	< 0.001^a^
CANVAS-program	Canagliflozin	0.86 (95% CI 0.75–0.97)	0.02

This is not a head-to-head comparison

*CVOT* cardiovascular outcomes trial, *MACE* major adverse cardiovascular events (cardiovascular death, non-fatal myocardial infarction or non-fatal stroke), *NS* not significant

^a^SUSTAIN™-6 was a non-inferiority study, and testing for superiority was not a pre-specified endpoint

#### Real world evidence studies

Currently, some agents such as exenatide and dapagliflozin only have CV data from meta-analyses or observational studies [45, 46]. Recently published results from the real-world evidence observational study CVD-REAL, in which the risk of HHF in individuals with T2D who had been newly initiated on SGLT2 inhibitors (canagliflozin, dapagliflozin or empagliflozin) was compared to other glucose-lowering drugs (oGLD), have shown similar trends for risk reductions in HF and all-cause mortality associated with SGLT2 inhibitor use [47].This held true for all six countries involved in the study, despite differences in the background treatment and the use of different SGLT2 inhibitors in the US and Europe. Furthermore, as both HF and the subclinical forms of HF are amongst the earliest vascular complications seen when following up diabetic patients who do not have CVD at baseline [48], it may be suggested that these patients in CVD-REAL benefited from the early use of SGLT2 inhibitors. Of note, there were clear differences between the outcomes in the propensity-matched CVD-REAL and the double-blind, randomised, placebo-controlled, CANVAS Program cohorts, despite canagliflozin comprising the largest overall component of the SGLT2 inhibitors in the CVD-REAL study [39, 47]. For example, in the CANVAS Program, although the combined primary endpoint of 3P-MACE was reduced by 14%, there was no significant reduction in all-cause mortality, yet in the CVD-REAL US cohort, where 75% of patients were receiving canagliflozin, mortality was reduced in the SGLT2 inhibitor group by 62% (HR 0.38, 95% CI 0.29–0.50) [39, 47, 49]. This is most likely to be as a result of the differing methodologies of the two studies and known limitations of using retrospective data. We therefore feel that although well-performed observational studies showing the same trend can lend support to results that have been obtained from RCTs, only prospective well-performed CVOTs, in which strict study criteria, equalisation of baseline characteristics, and elimination of observer bias, can offer robust evidence of CV benefit. Until the results of the dapagliflozin DECLARE CVOT study become available in 2018, we cannot be confident in a class effect for the SGLT2 inhibitor class. We currently advise that when using the treatment algorithms physicians should continue to evaluate each of the members of any of the classes on the basis of their individual efficacy and safety data [23, 33, 35–38].

#### Multidisciplinary management

A key aspect of therapy for patients with T2D and comorbidities is the provision of a multidisciplinary team, the members of which contribute in complementary ways to improving patient outcomes. CEEDEG recommended the specialities that should be included in the multidisciplinary team and suggested their key functions and responsibilities within the team (Table 3).

## Discussion

Patient outcomes are best served by the rapid implementation of emerging superior therapies, putting pressure on clinical guideline groups to continuously update their outputs. The addition of interim recommendation publications to be used in tandem with existing guidelines will enable a swifter response to a continuously changing therapeutic landscape. Recent data from CVOT studies of anti-diabetes drugs, and the anticipation of a stream of further CVOT results becoming available in the future, mean that the CEEDEG objective of providing regular companion recommendations is a welcome initiative.

By following a structured process, unbiased consensus treatment algorithms were derived that take into consideration data from CVOTs on the CV and renoprotective aspects of newer anti-diabetic agents and establish their place in the therapeutic landscape. This initiative interprets the data in such a way as to enable prescribing physicians to more easily determine the optimum treatment regimen for both the medical and lifestyle needs of their patients with T2D and CVD, or with T2D and CKD with or without CVD.

## Additional files

**Additional file 1.** Round 1 questionnaire.

**Additional file 2.** Initial recommendation scoring.

**Additional file 3.** Final recommendation scoring.

**Additional file 4: Table S1.** Selected outcomes from SGLT2 inhibitor CVOTs.

## Abbreviations

ACE: angiotensin-converting enzyme
ACR: albumin:creatinine ratio
ADA: American Diabetes Association
ARB: angiotensin receptor blocker
BMI: body mass index
BNP: brain natriuretic peptide
BP: blood pressure
CEEDEG: Central and Eastern European Diabetes Expert Group
CI: confidence interval
CKD: chronic kidney disease
CV: cardiovascular
CVD: cardiovascular disease
CVOT: cardiovascular outcomes trial
DPP-4: dipeptidyl peptidase 4
EASD: European Association for the Study of Diabetes
ECG: electrocardiogram
ECHO: echocardiogram
eGFR: estimated glomerular filtration rate
EMA: European Medicines Agency
ESC: European Society of Cardiology
FDA: US Food and Drug Administration
GFR: glomerular filtration rate
GLP-1: glucagon-like peptide 1
HbA1c: glycated haemoglobin A1c
HF: heart failure
HR: hazard ratio
LAR: long-acting release
MACE: major adverse cardiovascular events
MI: myocardial infarction
NS: not significant
NT-proBNP: N-terminal prohormone of BNP
ÖDG: Austrian Society for Diabetes
RAS: renin–angiotensin system
RRR: relative risk reduction
SGLT2: sodium–glucose co-transporter 2
SSED/SGED: Swiss Society of Endocrinology and Diabetes
SUs: sulfonylureas
T2D: type 2 diabetes
TZD: thiazolidinedione
UACR: urinary albumin/creatinine ratio
