# Supplementary material for: Translating recent results from the Cardiovascular Outcomes Trials into clinical practice: recommendations from the Central and Eastern European Diabetes Expert Group (CEEDEG)
Source: Cardiovasc Diabetol. 2017 Oct 23;16:137. doi: 10.1186/s12933-017-0622-7 (PMC5654048; doi:10.1186/s12933-017-0622-7)
Supplement: Supplementary file 1 — Additional file 1. Round 1 questionnaire. [file 12933_2017_622_MOESM1_ESM.pdf]

Cardiovascular Outcomes in Type 2 Diabetes – Updates to Treatment Approaches

Page 2: Determine the need for EASD/ADA guideline updates

Q1. Following the publication of positive results for liraglutide in the LEADER trial and empagliflozin in the EMPA-REG OUTCOME trial, should current EASD/ADA guidelines be updated?

Page 3: Please state how you believe EASD/ADA guidelines should be updated with regards to...

Q2. Patients with Type 2 Diabetes and established cardiovascular disease, at high risk of cardiovascular events**1 or more of: • history of myocardial infarction (MI)• evidence of multi-vessel coronary artery disease (CAD, significant stenosis or previous revascularisation)• evidence of single-vessel CAD plus positive non-invasive stress test for ischaemia or hospital discharge for unstable angina in past 12 months• unstable angina with presence of single- or multi-vessel CAD• history of stroke• occlusive peripheral artery disease

	Recommendation	Supporting comments
Assessment	-	-
Diagnosis	-	-
Management		-
Monitoring	-	-
Evaluation of response to treatment	-	-
Other	-	-

Page 4: Please state how you believe EASD/ADA guidelines should be updated with regards to...

Q3. Patients with Type 2 Diabetes and no established cardiovascular disease

	Recommendation	Supporting comments
Assessment		-
Diagnosis	-	-
Management		-
Monitoring	-	-
Evaluation of response to treatment	-	-
Other	-	-

Page 5: Please state how you believe EASD/ADA guidelines should be updated with regards to...

Q4. Patients with Type 2 Diabetes and established renal disease* *eGFR (MDRD) <60 mL/min/1.73 m2 and/or macroalbuminuria (urine albumin-to-creatinine ratio >300 mg/g

No Response

Page 6: Please state how you believe EASD/ADA guidelines should be updated with regards to...

Q5. Patients with Type 2 Diabetes and established renal* and cardiovascular disease***eGFR (MDRD) 300 mg/g**1 or more of: • history of myocardial infarction (MI)• evidence of multi-vessel coronary artery disease (CAD, significant stenosis or previous revascularisation)• evidence of single-vessel CAD plus positive non-invasive stress test for ischaemia or hospital discharge for unstable angina in past 12 months• unstable angina with presence of single- or multi-vessel CAD• history of stroke• occlusive peripheral artery disease

	Recommendation	Supporting comments
**Assessment**	-	-
**Diagnosis**	-	-
**Management**		-
**Monitoring**	-	-
**Evaluation of response to treatment**	-	-
**Other**	-	-

Page 7: Inter-professional, multidisciplinary team-based care

Q6. Please indicate how to improve long-term adherence rates and self-management in patients with Type 2 Diabetes and either cardiovascular or renal comorbidity.

No Response

Q7. Please indicate (and develop on how these integrate if needed) which specialties should be included to develop inter-professional, multidisciplinary team-based care for a comprehensive multifactorial risk-reduction strategy in the context of cardiovascular comorbidity.

Diabetologist/Endocrinologist

Internist

Nephrologist -

Nutritionist/Dietician -

Q7. Please indicate (and develop on how these integrate if needed) which specialties should be included to develop inter-professional, multidisciplinary team-based care for a comprehensive multifactorial risk-reduction strategy in the context of cardiovascular comorbidity.

**Pharmacist**	-
**Nurses**	-
**Certified diabetes educator (incl. smoking cessation)**	-

Q8. Please indicate (and develop on how these integrate if needed) which specialties should be included to develop inter-professional, multidisciplinary team-based care for a comprehensive multifactorial risk-reduction strategy in the context of renal comorbidity.

No Response
