# Supplementary material for: Translating recent results from the Cardiovascular Outcomes Trials into clinical practice: recommendations from the Central and Eastern European Diabetes Expert Group (CEEDEG)
Source: Cardiovasc Diabetol. 2017 Oct 23;16:137. doi: 10.1186/s12933-017-0622-7 (PMC5654048; doi:10.1186/s12933-017-0622-7)
Supplement: Supplementary file 2 — Additional file 2. Initial recommendation scoring. [file 12933_2017_622_MOESM2_ESM.docx]

**Cardiovascular Outcomes in Type 2 Diabetes – Updates to Treatment Approaches**

Objective for the proposed guideline topic:

To improve care for patients with type 2 diabetes and cardiovascular comorbidities.

Methodology

1. Review data from EMPA-REG OUTCOME and other cardiovascular outcome trials and consider their impact on current EASD/ADA guidelines
2. Using the Delphi process, achieve expert consensus on appropriate updates to the EASD/ADA guidelines
3. Following consensus, hold a live, half-day meeting with selected experts to review consensus guideline updates and draft instructions to practicing physicians to support their implementation
4. Recommendations, practical and clinical guidance to be published in an open-access journal

| QUESTIONNAIRE 1 |
| --- |

Following the publication of positive results from EMPA-REG OUTCOME and other cardiovascular outcome trial, should current EASD/ADA guidelines be updated? [Yes/No]

| **YES (9/9)** |
| --- |

| QUESTIONNAIRE 2 |
| --- |

The responses to Questionnaire 1 were sorted, de-duplicated, categorised, and used to create Questionnaire 2.

Task: Please score each of the recommendations, based on the degree to which you agree with them.

In addition, please feel free to add additional comments, suggestions and refinements against the proposed recommendations.

| Scoring system: |
| --- |
| [1] Totally disagree |
| [2] Disagree |
| [3] Unsure |
| [4] Agree |
| [5] Totally agree |

Consensus and exclusion criteria
Consensus criteria: 80% of respondents scoring 4, or 5
Exclusion criteria: 80% of respondents scoring 1, 2, or 3

A // Patients with Type 2 Diabetes and established cardiovascular disease, at high risk of cardiovascular events*

**1 or more of: history of myocardial infarction (MI), evidence of multi-vessel coronary artery disease (CAD, significant stenosis or previous revascularisation), evidence of single-vessel CAD plus positive non-invasive stress test for ischaemia or hospital discharge for unstable angina in past 12 months, unstable angina with presence of single- or multi-vessel CAD, history of stroke, occlusive peripheral artery disease*

| Recommendation | Scoring | Supporting comments | |
| --- | --- | --- | --- |
| Diagnosis / Assessments | | | |
| No change in diagnostic procedures to identify CVD is needed, however, these procedures should be implemented more frequently, especially in high-risk individuals | 1  2  3  4  5 |  | |
| Patients at high risk of CV events should be actively searched and screened | 1  2  3  4  5 |  | |
| - Initial assessment should comprise: | | | |
| - - Patient characteristics |  | |  |
| - - - Age | 1  2  3  4  5 | |  |
| - - - Sex | 1  2  3  4  5 | |  |
| - - - Height/Weight/BMI | 1  2  3  4  5 | |  |
| - - - Waist circumference | 1  2  3  4  5 | |  |
| - - - Ethnic group | 1  2  3  4  5 | |  |
| - - Medical history |  | |  |
| - - - Medical history in general | 1  2  3  4  5 | |  |
| - - - T2D duration | 1  2  3  4  5 | |  |
| - - - Prior hypoglycemia | 1  2  3  4  5 | |  |
| - - - History or concurrent disease | 1  2  3  4  5 | |  |
| - - - - Pancreatitis | 1  2  3  4  5 | |  |
| - - - - Medullary cancer | 1  2  3  4  5 | |  |
| - - - - Genital infection | 1  2  3  4  5 | |  |
| - - Laboratory values/exploration: |  | |  |
| - - - BNP/NT-proBNP | 1  2  3  4  5 | |  |
| - - - HbA1C | 1  2  3  4  5 | |  |
| - - - Lipid profile | 1  2  3  4  5 | |  |
| - - - Proteinuria | 1  2  3  4  5 | |  |
| - - - GFR/eGFR | 1  2  3  4  5 | |  |
| - - - Albumin:creatinine ratio (ACR) | 1  2  3  4  5 | |  |
| - - Clinical examination |  | |  |
| - - - BP | 1  2  3  4  5 | |  |
| - - - NYHA class of heart failure | 1  2  3  4  5 | |  |
| - - - Risk of infection | 1  2  3  4  5 | |  |
| - - In patients at high risk of myocardial infarction |  | |  |
| - - - ECG | 1  2  3  4  5 | |  |
| - - - Cardiac ultrasound | 1  2  3  4  5 | |  |
| - - - Abdominal ultrasound | 1  2  3  4  5 | |  |
| - - - Angiography | 1  2  3  4  5 | |  |
| - - The influence of current therapies should be considered | 1  2  3  4  5 | |  |
| - - The influence of current comorbidities should be considered | 1  2  3  4  5 | |  |

| Recommendation | Scoring | Supporting comments |
| --- | --- | --- |
| Management | | |
| - A treat-to target approach should be conducted: |  |  |
| - - HbA1c about 7% // 53 mmol/M *(note: ADA and EASD targets are different)* | 1  2  3  4  5 |  |
| - - For fragile patients: 7,5% // 60 mmol/M or even higher | 1  2  3  4  5 |  |
| - The management of T2D patients with CVD is a complex approach that should comprise antihyperglycaemic therapy with: |  |  |
| - - Metformin | 1  2  3  4  5 |  |
| - - - - monotherapy with metformin should no more be used except of excellent diabetes control (glucose in target range) | 1  2  3  4  5 |  |
| - - - - combination therapy should be based on individual patient’s data *and is necessary to maintain good glycemic control with respect to the long-term diabetes complications in case of longer survival due to reduced CV/total death rate* | 1  2  3  4  5 |  |
| - - SGLT2 inhibitor (empagliflozin) or GLP-1 RA (liraglutide) should be considered as second line therapy after metformin | 1  2  3  4  5 |  |
| - - SGLT2 inhibitor (empagliflozin) | 1  2  3  4  5 |  |
| - - - - preferentially for patients with heart failure but not with history of stroke | 1  2  3  4  5 |  |
| - - - - if GFR >60 ml/min | 1  2  3  4  5 |  |
| - - - - always used as a second line therapy WITH metformin | 1  2  3  4  5 |  |
| - - - - as it has a more profound effect on reduction of CV risk and is non-injectable, it should be preferred to liraglutide in most cases | 1  2  3  4  5 |  |
| - - - - as a first line of antidiabetic therapy in case of intolerance or contraindications to metformin | 1  2  3  4  5 |  |
| - - If GFR< 60 ml/min, therapy with DPP4, liraglutide or insulin should be considered | 1  2  3  4  5 |  |
| - - GLP-1 RA (liraglutide) | 1  2  3  4  5 |  |
| - - - - should always be used as a second line therapy WITH metformin | 1  2  3  4  5 |  |
| - - - - should be considered in patients with BMI>30 kg.m^2^ and already treated with insulin | 1  2  3  4  5 |  |
| - - - - can be considered as a first line antidiabetic therapy in case of intolerance or contraindications to metformin | 1  2  3  4  5 |  |
| - Non-recommended therapies |  |  |
| - - These patients should not be treated with thiazolidinediones or sulphonylureas (except of gliclazide) *– Use of sulphonylurea consistently yields increased CV risk in these patients and there is a severe risk that it can mitigate or even override the positive effects of empagliflozin/liraglutide.* | 1  2  3  4  5 |  |
| - Patients should also receive | | |
| - - Aspirin therapy | 1  2  3  4  5 |  |
| - - Beta-blocker therapy | 1  2  3  4  5 |  |
| - - Angiotensin-converting enzyme inhibitor therapy | 1  2  3  4  5 |  |
| - - Statin therapy | 1  2  3  4  5 |  |
| - Patient’s age should be considered | 1  2  3  4  5 |  |
| - Patient’s comorbidities should be considered | 1  2  3  4  5 |  |
| - Patient’s preferences should be considered | 1  2  3  4  5 |  |

| Recommendation | Scoring | Supporting comments |
| --- | --- | --- |
| Monitoring / Evaluation of response to treatment | | |
| - Monitoring for efficacy of glucose-lowering agents is necessary | 1  2  3  4  5 |  |
| - Monitoring should be conducted every 3 months | 1  2  3  4  5 |  |
| - Clinical examination should comprise: |  |  |
| - - Blood pressure | 1  2  3  4  5 |  |
| - - Height/Weight/BMI | 1  2  3  4  5 |  |
| - - Waist circumference | 1  2  3  4  5 |  |
| - - Calorie intake | 1  2  3  4  5 |  |
| - Laboratory values: |  |  |
| - - Glucose levels (correction of insulin/antihypertensive drugs if hypoglycaemia) | 1  2  3  4  5 |  |
| - - HbA1C | 1  2  3  4  5 |  |
| - - Lipid profile | 1  2  3  4  5 |  |
| - - Proteinuria | 1  2  3  4  5 |  |
| - - GFR/eGFR | 1  2  3  4  5 |  |
| - - - stop when GFR < 45 ml/min for empagliflozin | 1  2  3  4  5 |  |
| - - - stop when GFR < 30 ml/min for liraglutide | 1  2  3  4  5 |  |
| - - Albumin:creatinine ratio (ACR) | 1  2  3  4  5 |  |
| - - Insulin dosage | 1  2  3  4  5 |  |
| - - In patients receiving empagliflozin: ketonuria/ketonemia (ketoacidosis) | 1  2  3  4  5 |  |
| - Imaging/exploration: |  |  |
| - - ECG | 1  2  3  4  5 |  |
| - - Abdominal ultrasound (gallstone formation) | 1  2  3  4  5 |  |
| - Non-responders (glucose lowering) to empagliflozin should not be simply switched to liraglutide | 1  2  3  4  5 |  |
| - - Reduction of HF and renoprotective effects of empagliflozin should be considered prior to empagliflozin discontinuation | 1  2  3  4  5 |  |
| - - Dual therapy with empagliflozin/liraglutide should be considered first | 1  2  3  4  5 |  |

B // Patients with Type 2 Diabetes and no established cardiovascular disease

| Recommendation | Scoring | Supporting comments |
| --- | --- | --- |
| Diagnosis / Assessments | | |
| - Patients with CVD should be actively screened | 1  2  3  4  5 |  |
| - Initial assessment should comprise: | | |
| - - Patient characteristics |  |  |
| - - - Age | 1  2  3  4  5 |  |
| - - - Sex | 1  2  3  4  5 |  |
| - - - Height/Weight/BMI | 1  2  3  4  5 |  |
| - - - Waist circumference | 1  2  3  4  5 |  |
| - - - Ethnic group | 1  2  3  4  5 |  |
| - - Medical history |  |  |
| - - - Medical history in general | 1  2  3  4  5 |  |
| - - - T2D duration | 1  2  3  4  5 |  |
| - - - Prior hypoglycemia | 1  2  3  4  5 |  |
| - - - History or concurrent disease | 1  2  3  4  5 |  |
| - - - - Pancreatitis | 1  2  3  4  5 |  |
| - - - - Medullary cancer | 1  2  3  4  5 |  |
| - - - - Genital infection | 1  2  3  4  5 |  |
| - - Laboratory values/exploration: |  |  |
| - - - BNP/NT-proBNP | 1  2  3  4  5 |  |
| - - - HbA1C | 1  2  3  4  5 |  |
| - - - Lipid profile | 1  2  3  4  5 |  |
| - - - Proteinuria | 1  2  3  4  5 |  |
| - - - GFR/eGFR | 1  2  3  4  5 |  |
| - - - Albumin:creatinine ratio (ACR) | 1  2  3  4  5 |  |
| - - Clinical examination |  |  |
| - - - Blood pressure | 1  2  3  4  5 |  |
| - - - Ankle-brachial (ABI) index | 1  2  3  4  5 |  |
| - - - NYHA class of HF | 1  2  3  4  5 |  |
| - - - Risk of infection | 1  2  3  4  5 |  |
| - - Imaging/Exploration |  |  |
| - - - ECG | 1  2  3  4  5 |  |
| - - - Cardiac ultrasound | 1  2  3  4  5 |  |
| - - - Abdominal ultrasound | 1  2  3  4  5 |  |
| - - - Cardiac CT/calcium scoring | 1  2  3  4  5 |  |
| - - - Angiography | 1  2  3  4  5 |  |
| - - The influence of current therapies should be considered | 1  2  3  4  5 |  |
| - - The influence of comorbidities should be considered | 1  2  3  4  5 |  |

| Recommendation | Scoring | Supporting comments | |
| --- | --- | --- | --- |
| Management | | | |
| - A treat-to target approach should be conducted: |  | |  |
| - - HbA1c target below 7% | 1  2  3  4  5 | |  |
| - - HbA1c target below 6.5% | 1  2  3  4  5 | |  |
| - The management of T2D patients should be conducted as in the current guidelines | 1  2  3  4  5 | |  |
| - - Metformin | 1  2  3  4  5 | |  |
| - - - - monotherapy with metformin should no more be used except of excellent diabetes control (glucose in target range) | 1  2  3  4  5 | |  |
| - - - - combination therapy should be based on individual patient’s data *and is necessary to maintain good glycemic control with respect to the long-term diabetes complications in case of longer survival due to reduced CV/total death rate* | 1  2  3  4  5 | |  |
| - - Other classes |  | |  |
| - - - Pioglitazone if normal renal function | 1  2  3  4  5 | |  |
| - - - Gliptins in elderly patients with risk of hypoglycaemia | 1  2  3  4  5 | |  |
| - - - If GFR< 60 ml/min, therapy with gliptins, liraglutide or insulin should be considered | 1  2  3  4  5 | |  |
| - New data on empagliflozin and liraglutide: as second line? |  | |  |
| - - SGLT2 inhibitor (empagliflozin) or GLP-1 RA (liraglutide) should be considered as second line therapy after metformin, if necessary and possible   *Note from one of the advisors: Use of empagliflozin / liraglutide will make no harm to these patients and in patients with silent CVD it may be even beneficial. There is no reason why not to recommend empa/lira as a second line after metformin.* | 1  2  3  4  5 | |  |
| - - Empagliflozin or liraglutide may be an option in patients with BMI>30, with required weight loss | 1  2  3  4  5 | |  |
| - - SGLT2 inhibitor (empagliflozin) | 1  2  3  4  5 | |  |
| - - - - should always be used as a second line therapy WITH metformin | 1  2  3  4  5 | |  |
| - - - - as a first line of antidiabetic therapy in case of intolerance or contraindications to metformin | 1  2  3  4  5 | |  |
| - - GLP-1 RA (liraglutide) | 1  2  3  4  5 | |  |
| - - - - should always be used as a second line therapy WITH metformin | 1  2  3  4  5 | |  |
| - - - - can be considered as a first line antidiabetic therapy in case of intolerance or contraindications to metformin | 1  2  3  4  5 | |  |
| - Patients should also receive, if necessary | | | |
| - - Aspirin therapy | 1  2  3  4  5 | |  |
| - - Antihypertensive therapy | 1  2  3  4  5 | |  |
| - - Statin therapy | 1  2  3  4  5 | |  |
| - Patient’s age should be considered | 1  2  3  4  5 | |  |
| - Patient’s preferences should be considered | 1  2  3  4  5 | |  |
| - Treatment selection should be done according to comorbidities and possible adverse events | 1  2  3  4  5 | |  |

| Recommendation | Scoring | Supporting comments |
| --- | --- | --- |
| Monitoring / Evaluation of response to treatment | | |
| - Monitoring for efficacy of glucose-lowering agents is necessary | 1  2  3  4  5 |  |
| - Monitoring should be conducted every 3 months   *Note from one of the advisors: As these patients are usually younger, more intensive monitoring should be established which can help to maintain more stringent diabetes control.* | 1  2  3  4  5 |  |
| - Clinical examination should comprise: |  |  |
| - - Blood pressure | 1  2  3  4  5 |  |
| - - Height/Weight/BMI | 1  2  3  4  5 |  |
| - - Waist circumference | 1  2  3  4  5 |  |
| - - Calorie intake | 1  2  3  4  5 |  |
| - Laboratory values: |  |  |
| - - Glucose levels (correction of insulin/antihypertensive drugs if hypoglycaemia) | 1  2  3  4  5 |  |
| - - HbA1C | 1  2  3  4  5 |  |
| - - Lipid profile | 1  2  3  4  5 |  |
| - - Proteinuria | 1  2  3  4  5 |  |
| - - GFR/eGFR | 1  2  3  4  5 |  |
| - - - stop when GFR < 45 ml/min for empagliflozin | 1  2  3  4  5 |  |
| - - - stop when GFR < 30 ml/min for liraglutide | 1  2  3  4  5 |  |
| - - albumin:creatinine ratio (ACR) | 1  2  3  4  5 |  |
| - - Insulin dosage | 1  2  3  4  5 |  |
| - - In patients receiving empagliflozin: ketonuria/ketonemia (ketoacidosis) | 1  2  3  4  5 |  |
| - Imaging/exploration: |  |  |
| - - ECG | 1  2  3  4  5 |  |
| - - Cardiac ultrasound | 1  2  3  4  5 |  |
| - - Abdominal ultrasound (gallstone formation) | 1  2  3  4  5 |  |
| - Non-responders (glucose lowering) to empagliflozin should not be simply switched to liraglutide | 1  2  3  4  5 |  |
| - - Renoprotective effects of empagliflozin should be considered prior to empagliflozin discontinuation | 1  2  3  4  5 |  |
| - - Dual therapy with empagliflozin/liraglutide should be considered first | 1  2  3  4  5 |  |

C // Patients with Type 2 Diabetes and established renal disease*

*** *eGFR (MDRD) <60 mL/min/1.73 m2 and/or macroalbuminuria (urine albumin-to-creatinine ratio >300 mg/g.*

| Recommendation | Scoring | Supporting comments |
| --- | --- | --- |
| Diagnosis / Assessments | | |
| - Patients at high risk of CV events should be actively searched and screened in renal disease patients | 1  2  3  4  5 |  |
| - Initial assessment should comprise: | | |
| - - Patient characteristics |  |  |
| - - - Age | 1  2  3  4  5 |  |
| - - - Sex | 1  2  3  4  5 |  |
| - - - Height/Weight/BMI | 1  2  3  4  5 |  |
| - - - Waist circumference | 1  2  3  4  5 |  |
| - - - Ethnic group | 1  2  3  4  5 |  |
| - - Medical history |  |  |
| - - - Medical history in general | 1  2  3  4  5 |  |
| - - - T2D duration | 1  2  3  4  5 |  |
| - - - Prior hypoglycemia | 1  2  3  4  5 |  |
| - - - History or concurrent disease | 1  2  3  4  5 |  |
| - - - - Pancreatitis | 1  2  3  4  5 |  |
| - - - - Medullary cancer | 1  2  3  4  5 |  |
| - - - - Genital infection | 1  2  3  4  5 |  |
| - - - Co-morbidities/renal complications | 1  2  3  4  5 |  |
| - - - - retinopathy | 1  2  3  4  5 |  |
| - - - - haematological disorders | 1  2  3  4  5 |  |
| - - Laboratory values/exploration: |  |  |
| - - - BNP/NT-proBNP | 1  2  3  4  5 |  |
| - - - HbA1C | 1  2  3  4  5 |  |
| - - - Lipid profile | 1  2  3  4  5 |  |
| - - - Proteinuria | 1  2  3  4  5 |  |
| - - - GFR/eGFR | 1  2  3  4  5 |  |
| - - - Albumin:creatinine ratio (ACR) | 1  2  3  4  5 |  |
| - - - Urinalysis | 1  2  3  4  5 |  |
| - - - Ruling out other causes/kidney diseases associated with macroalbuminuria, incl. the detection of nondiabetic nephropathy | 1  2  3  4  5 |  |
| - - Clinical examination |  |  |
| - - - BP | 1  2  3  4  5 |  |
| - - - NYHA class of HF | 1  2  3  4  5 |  |
| - - - Risk of infection | 1  2  3  4  5 |  |
| - - Imaging/exploration |  |  |
| - - - ECG | 1  2  3  4  5 |  |
| - - - Cardiac ultrasound | 1  2  3  4  5 |  |
| - - - Abdominal ultrasound | 1  2  3  4  5 |  |
| - - - Kidney ultrasound | 1  2  3  4  5 |  |
| - - - Angiography | 1  2  3  4  5 |  |
| - - The influence of current therapies should be considered | 1  2  3  4  5 |  |
| - - The influence of comorbidities should be considered | 1  2  3  4  5 |  |

| Recommendation | Scoring | Supporting comments |
| --- | --- | --- |
| Management | | |
| - A treat-to target approach should be conducted: |  |  |
| - - HbA1c target of about 7% // 53 mmol/M *(note: ADA and EASD targets are different)* | 1  2  3  4  5 |  |
| - The management of T2D patients with renal disease is a complex approach that should comprise antihyperglycaemic therapy with: |  |  |
| - - Metformin | 1  2  3  4  5 |  |
| - - - - monotherapy with metformin should no more be used except of excellent diabetes control (glucose in target range) | 1  2  3  4  5 |  |
| - - - - combination therapy should be based on individual patient’s data *and is necessary to maintain good glycemic control with respect to the long-term diabetes complications in case of longer survival due to reduced CV/total death rate* | 1  2  3  4  5 |  |
| - - SGLT2 inhibitor (empagliflozin) should be considered as first line therapy with metformin | 1  2  3  4  5 |  |
| - - - In addition to RAS blockade | 1  2  3  4  5 |  |
| - - - - as it can reduce the rate of decline in renal function | 1  2  3  4  5 |  |
| - - - - if GFR >45 ml/min | 1  2  3  4  5 |  |
| - - - - as it has a more profound effect on reduction of CV risk and is non-injectable, it should be preferred to liraglutide in most cases | 1  2  3  4  5 |  |
| - - - - as monotherapy in case of intolerance or contraindications to metformin | 1  2  3  4  5 |  |
| - - If GFR< 15 ml/min, therapy with DPP4, liraglutide or insulin should be considered | 1  2  3  4  5 |  |
| - - GLP-1 RA (liraglutide) | 1  2  3  4  5 |  |
| - - - - should always be used as a second line therapy WITH metformin | 1  2  3  4  5 |  |
| - - - - should be considered in patients with BMI>30 kg.m^2^ and already treated with insulin | 1  2  3  4  5 |  |
| - - - - can be considered as a first line antidiabetic therapy in case of intolerance or contraindications to metformin | 1  2  3  4  5 |  |
| - Non-recommended therapies |  |  |
| - - These patients should not be treated with thiazolidinediones or sulphonylureas (except of gliclazide) *– Use of sulphonylurea consistently yields increased CV risk in these patients and there is a severe risk that it can mitigate or even override the positive effects of empagliflozin/liraglutide.* | 1  2  3  4  5 |  |
| - Patients should also receive | | |
| - - Aspirin therapy | 1  2  3  4  5 |  |
| - - Beta-blocker therapy | 1  2  3  4  5 |  |
| - - ACE/ARB therapy | 1  2  3  4  5 |  |
| - - Diuretics | 1  2  3  4  5 |  |
| - Patient’s age should be considered | 1  2  3  4  5 |  |
| - Patient’s comorbidities should be considered | 1  2  3  4  5 |  |
| - Patient’s preferences should be considered | 1  2  3  4  5 |  |

| Recommendation | Scoring | Supporting comments |
| --- | --- | --- |
| Monitoring / Evaluation of response to treatment | | |
| - Monitoring for efficacy of glucose-lowering agents is necessary | 1  2  3  4  5 |  |
| - Monitoring should be conducted every 3 months | 1  2  3  4  5 |  |
| - Clinical examination should comprise: |  |  |
| - - Blood pressure (should be below 130/85 mmHg) | 1  2  3  4  5 |  |
| - - Height/Weight/BMI | 1  2  3  4  5 |  |
| - - Waist circumference | 1  2  3  4  5 |  |
| - - Calorie intake | 1  2  3  4  5 |  |
| - Laboratory values: |  |  |
| - - Glucose levels (correction of insulin/antihypertensive drugs if hypoglycaemia) | 1  2  3  4  5 |  |
| - - HbA1C | 1  2  3  4  5 |  |
| - - Albumin-corrected fructosamine | 1  2  3  4  5 |  |
| - - Lipid profile (LDL should be below 70 mg/dl) | 1  2  3  4  5 |  |
| - - Proteinuria | 1  2  3  4  5 |  |
| - - GFR/eGFR (at least twice a year) | 1  2  3  4  5 |  |
| - - - stop when GFR < 45 ml/min for empagliflozin | 1  2  3  4  5 |  |
| - - - stop when GFR < 30 ml/min for liraglutide | 1  2  3  4  5 |  |
| - - Albumin:creatinine ratio (ACR) | 1  2  3  4  5 |  |
| - - Assessment of renal function, necessity of dialysis | 1  2  3  4  5 |  |
| - - Insulin dosage | 1  2  3  4  5 |  |
| - - In patients receiving empagliflozin: ketonuria/ketonemia (ketoacidosis) | 1  2  3  4  5 |  |
| - Clinical examination |  |  |
| - - Haematology probrem | 1  2  3  4  5 |  |
| - - Anemia | 1  2  3  4  5 |  |
| - Imaging/exploration: |  |  |
| - - ECG | 1  2  3  4  5 |  |
| - - Abdominal ultrasound (gallstone formation) | 1  2  3  4  5 |  |
| - Non-responders (glucose lowering) to empagliflozin should not be simply switched to liraglutide | 1  2  3  4  5 |  |
| - - Reduction of HF and renoprotective effects of empagliflozin should be considered prior to empagliflozin discontinuation | 1  2  3  4  5 |  |
| - - Dual therapy with empagliflozin/liraglutide should be considered first | 1  2  3  4  5 |  |

D // Patients with Type 2 Diabetes and established renal* and cardiovascular disease**

**1 or more of: history of myocardial infarction (MI), evidence of multi-vessel coronary artery disease (CAD, significant stenosis or previous revascularisation), evidence of single-vessel CAD plus positive non-invasive stress test for ischaemia or hospital discharge for unstable angina in past 12 months, unstable angina with presence of single- or multi-vessel CAD, history of stroke, occlusive peripheral artery disease. **eGFR (MDRD) <60 mL/min/1.73 m2 and/or macroalbuminuria (urine albumin-to-creatinine ratio >300 mg/g.*

| Recommendation | Scoring | Supporting comments |
| --- | --- | --- |
| Diagnosis / Assessments | | |
| - No change in diagnostic procedures to identify CVD is needed, however, these procedures should be implemented more frequently, especially in high-risk individuals. | 1  2  3  4  5 |  |
| - Patients at high risk of CV events should be actively searched and screened | 1  2  3  4  5 |  |
| - Initial assessment should comprise: | | |
| - - Patient characteristics |  |  |
| - - - Age | 1  2  3  4  5 |  |
| - - - Sex | 1  2  3  4  5 |  |
| - - - Height/Weight/BMI | 1  2  3  4  5 |  |
| - - - Waist circumference | 1  2  3  4  5 |  |
| - - - Ethnic group | 1  2  3  4  5 |  |
| - - Medical history |  |  |
| - - - Medical history in general | 1  2  3  4  5 |  |
| - - - T2D duration | 1  2  3  4  5 |  |
| - - - Prior hypoglycemia | 1  2  3  4  5 |  |
| - - - History or concurrent disease | 1  2  3  4  5 |  |
| - - - - Pancreatitis | 1  2  3  4  5 |  |
| - - - - Medullary cancer | 1  2  3  4  5 |  |
| - - - - Genital infection | 1  2  3  4  5 |  |
| - - Laboratory values/exploration: |  |  |
| - - - BNP/NT-proBNP | 1  2  3  4  5 |  |
| - - - HbA1C | 1  2  3  4  5 |  |
| - - - Lipid profile | 1  2  3  4  5 |  |
| - - - Proteinuria | 1  2  3  4  5 |  |
| - - - GFR/eGFR | 1  2  3  4  5 |  |
| - - - Albumin:creatinine ratio (ACR) | 1  2  3  4  5 |  |
| - - - Ruling out other causes/kidney diseases associated with macroalbuminuria, incl. the detection of nondiabetic nephropathy | 1  2  3  4  5 |  |
| - - Clinical examination |  |  |
| - - - BP | 1  2  3  4  5 |  |
| - - - NYHA class of HF | 1  2  3  4  5 |  |
| - - - Risk of infection | 1  2  3  4  5 |  |
| - - In patients at high-risk for MI |  |  |
| - - - ECG | 1  2  3  4  5 |  |
| - - - Cardiac ultrasound | 1  2  3  4  5 |  |
| - - - Abdominal ultrasound | 1  2  3  4  5 |  |
| - - - Kidney ultrasound | 1  2  3  4  5 |  |
| - - - Angiography | 1  2  3  4  5 |  |
| - - The influence of current therapies should be considered | 1  2  3  4  5 |  |
| - - The influence of comorbidities should be considered | 1  2  3  4  5 |  |

| Recommendation | Scoring | Supporting comments |
| --- | --- | --- |
| Management | | |
| - A treat-to target approach should be conducted: |  |  |
| - - HbA1c about 7% // 53 mmol/M *(note: ADA and EASD targets are different)* | 1  2  3  4  5 |  |
| - - For fragile patients: 7,5% // 60 mmol/M or even higher | 1  2  3  4  5 |  |
| - The management of T2D patients with CVD is a complex approach that should comprise antihyperglycaemic therapy with: |  |  |
| - - Metformin | 1  2  3  4  5 |  |
| - - - - monotherapy with metformin should no more be used except of excellent diabetes control (glucose in target range) | 1  2  3  4  5 |  |
| - - - - combination therapy should be based on individual patient’s data *and is necessary to maintain good glycemic control with respect to the long-term diabetes complications in case of longer survival due to reduced CV/total death rate* | 1  2  3  4  5 |  |
| - - SGLT2 inhibitor (empagliflozin) should be considered as first line therapy after metformin | 1  2  3  4  5 |  |
| - - - - as it can reduce the rate of decline in renal function | 1  2  3  4  5 |  |
| - - - - preferentially for patients with heart failure but not with history of stroke | 1  2  3  4  5 |  |
| - - - - if GFR >45 ml/min | 1  2  3  4  5 |  |
| - - - - if GFR >30 ml/min | 1  2  3  4  5 |  |
| - - - - as it has a more profound effect on reduction of CV risk and is non-injectable, it should be preferred to liraglutide in most cases | 1  2  3  4  5 |  |
| - - - - as monotherapy in case of intolerance or contraindications to metformin | 1  2  3  4  5 |  |
| - - SGLT2 inhibitor (empagliflozin) as second line therapy in combination with liraglutide | 1  2  3  4  5 |  |
| - - If GFR< 60 ml/min, therapy with DPP4, liraglutide or insulin should be considered | 1  2  3  4  5 |  |
| - - GLP-1 RA (liraglutide) | 1  2  3  4  5 |  |
| - - - - should always be used as a second line therapy WITH metformin | 1  2  3  4  5 |  |
| - - - - should be considered in patients with BMI>30 kg.m^2^ and already treated with insulin | 1  2  3  4  5 |  |
| - - - - can be considered as a first line antidiabetic therapy in case of intolerance or contraindications to metformin | 1  2  3  4  5 |  |
| - Non-recommended therapies |  |  |
| - - These patients should not be treated with thiazolidinediones or sulphonylureas (except of gliclazide) *– Use of sulphonylurea consistently yields increased CV risk in these patients and there is a severe risk that it can mitigate or even override the positive effects of empagliflozin/liraglutide.* | 1  2  3  4  5 |  |
| - Patients should also receive | | |
| - - Aspirin therapy | 1  2  3  4  5 |  |
| - - Beta-blocker therapy | 1  2  3  4  5 |  |
| - - ACE/ARB therapy | 1  2  3  4  5 |  |
| - - Statin therapy | 1  2  3  4  5 |  |
| - - Diuretics | 1  2  3  4  5 |  |
| - Patient’s age should be considered | 1  2  3  4  5 |  |
| - Patient’s comorbidities should be considered | 1  2  3  4  5 |  |
| - Patient’s preferences should be considered | 1  2  3  4  5 |  |

| Recommendation | Scoring | Supporting comments |
| --- | --- | --- |
| Monitoring / Evaluation of response to treatment | | |
| - Monitoring for efficacy of glucose-lowering agents is necessary | 1  2  3  4  5 |  |
| - Monitoring should be conducted every 3 months | 1  2  3  4  5 |  |
| - Regular control visits should be organized | 1  2  3  4  5 |  |
| - Clinical examination should comprise: |  |  |
| - - Blood pressure | 1  2  3  4  5 |  |
| - - Height/Weight/BMI | 1  2  3  4  5 |  |
| - - Waist circumference | 1  2  3  4  5 |  |
| - - Calorie intake | 1  2  3  4  5 |  |
| - Laboratory values: |  |  |
| - - Glucose levels (correction of insulin/antihypertensive drugs if hypoglycaemia) | 1  2  3  4  5 |  |
| - - HbA1C | 1  2  3  4  5 |  |
| - - albumin-corrected fructosamine | 1  2  3  4  5 |  |
| - - Lipid profile | 1  2  3  4  5 |  |
| - - Proteinuria | 1  2  3  4  5 |  |
| - - GFR/eGFR | 1  2  3  4  5 |  |
| - - - stop when GFR < 45 ml/min for empagliflozin | 1  2  3  4  5 |  |
| - - - stop when GFR < 30 ml/min for liraglutide | 1  2  3  4  5 |  |
| - - albumin:creatinine ratio (ACR) | 1  2  3  4  5 |  |
| - - assessment of renal function, necessity of dialysis | 1  2  3  4  5 |  |
| - - Insulin dosage | 1  2  3  4  5 |  |
| - - In patients receiving empagliflozin: ketonuria/ketonemia (ketoacidosis) | 1  2  3  4  5 |  |
| - Imaging/exploration: |  |  |
| - - ECG | 1  2  3  4  5 |  |
| - - Abdominal ultrasound (gallstone formation) | 1  2  3  4  5 |  |
| - Non-responders (glucose lowering) to empagliflozin should not be simply switched to liraglutide | 1  2  3  4  5 |  |
| - - Reduction of HF and renoprotective effects of empagliflozin should be considered prior to empagliflozin discontinuation | 1  2  3  4  5 |  |
| - - Dual therapy with empagliflozin/liraglutide should be considered first | 1  2  3  4  5 |  |

E // How to improve long-term adherence rates and self-management in patients with T2D

and either cardiovascular or renal comorbidity

| Recommendation | Scoring | Supporting comments |
| --- | --- | --- |
| - Clinical practice |  |  |
| - - Treatment strategies and dosing regimens should be simplified to improve medication adherence | 1  2  3  4  5 |  |
| - - Disease management programs should be implemented, and run by both specialists and GPs | 1  2  3  4  5 |  |
| - - Treatment strategies should be behavioral patient-centered strategies and care, with decision-making done within the context of patient priorities and goals | 1  2  3  4  5 |  |
| - Patient education |  |  |
| - - Treatment benefits should be better explained to patients | 1  2  3  4  5 |  |
| - - Careful, thorough, continuous and repeated patient education should be conducted | 1  2  3  4  5 |  |
| - - Patient education should be consistent throughout different health care practitioners | 1  2  3  4  5 |  |
| - - Patient education should involved a trained dietician and, if necessary, a psychologist | 1  2  3  4  5 |  |

F // Specialties that should be included to develop inter-professional, multidisciplinary team-based T2D care for a comprehensive multifactorial risk-reduction strategy in the context of cardiovascular comorbidity

| Recommendation | Notes from the advisors | Scoring | Supporting comments |
| --- | --- | --- | --- |
| Clinical specialties |  |  |  |
| Diabetologist / Endocrinologist | For therapy induction  Should be better trained in cardiology/nephrology/internal medicine  Leading figure within team in charge of education and guideline development for T2M patients  BP, lipid, HbA1c targets and control visits  HbA1C targets, explanation of therapy choice, possible side effects, complications of DM, acute emergencies | 1  2  3  4  5 |  |
| Internist | For follow-up  BP, lipid targets and controls, ECG, ECHO  Should be trained in diabetology, however, complicated diabetic patients should always be referred to diabetologists/endocrinologists | 1  2  3  4  5 |  |
| Nephrologist | Renal function, ACR, BP control  Lipid targets | 1  2  3  4  5 |  |
| Cardiologist | Key for CV complications and risk management  Should be better trained in diabetology | 1  2  3  4  5 |  |
| Other key actors |  |  |  |
| Nutritionist/Dietician | Dietary counseling at therapy induction  A keystone in patient education  Glucose control and intake, body weight  Individual diet plans  Weight reduction (if needed), long term maintenance | 1  2  3  4  5 |  |
| Pharmacist | Advise on use of therapies, detection of side effects, patient education | 1  2  3  4  5 |  |
| Nurses | Education programs, individual support, BMI, weight/height measures | 1  2  3  4  5 |  |
| Certified diabetes educator (incl. smoking cessation) | Diabetes education at treatment induction and for re-enforcement  Education programs, team work  They should guarantee consistent and structured education which is heavily underestimated worldwide, Europe included  Crucial for adherence, barriers to treatment, motivation | 1  2  3  4  5 |  |

G // Specialties that should be included to develop inter-professional, multidisciplinary team-based T2D care for a comprehensive multifactorial risk-reduction strategy in the context of renal comorbidity

| Recommendation | Notes from the advisors | Scoring | Supporting comments |
| --- | --- | --- | --- |
| Clinical specialties |  |  |  |
| Diabetologist / Endocrinologist | For therapy induction  Should be better trained in cardiology/nephrology/internal medicine  Leading figure within team in charge of education and guideline development for T2M patients  BP, lipid, HbA1c targets and control visits  HbA1C targets, explanation of therapy choice, possible side effects, complications of DM, acute emergencies | 1  2  3  4  5 |  |
| Internist | For follow-up  BP, lipid targets and controls  Should be trained in diabetology, however, complicated diabetic patients should always be referred to diabetologists/endocrinologists | 1  2  3  4  5 |  |
| Nephrologist | Renal function, ACR, BP control  CKD management and lipid targets | 1  2  3  4  5 |  |
| Cardiologist | Should be better trained in diabetology | 1  2  3  4  5 |  |
| Other key actors |  |  |  |
| Nutritionist/Dietician | Dietary counseling at therapy induction  A keystone in patient education  Glucose control and intake, body weight  Individual diet plans  Weight reduction (if needed), long term maintenance | 1  2  3  4  5 |  |
| Pharmacist | Advise on use of therapies, detection of side effects, patient education | 1  2  3  4  5 |  |
| Nurses | Education programs, individual support, BMI, weight/height measures | 1  2  3  4  5 |  |
| Certified diabetes educator (incl. smoking cessation) | Diabetes education at treatment induction and for re-enforcement  Education programs, team work  They should guarantee consistent and structured education which is heavily underestimated worldwide, Europe included  Crucial for adherence, barriers to treatment, motivation | 1  2  3  4  5 |  |

**You have completed the second questionnaire!**

Thank you for your participation in the Delphi process!

Your responses and those of your expert colleagues will be sorted, de-duplicated, categorised, and used to create Questionnaire 3.

Questionnaire 3 will be with you shortly. Deadline for completing Questionnaire 2 is **8 September**.

If you have any questions or concerns, please contact Caroline Charles or Claire Péan at [caroline.charles@fortispharma.com](mailto:caroline.charles@fortispharma.com) / [claire.pean@fortispharma.com](mailto:claire.pean@fortispharma.com)
