# Supplementary material for: Translating recent results from the Cardiovascular Outcomes Trials into clinical practice: recommendations from the Central and Eastern European Diabetes Expert Group (CEEDEG)
Source: Cardiovasc Diabetol. 2017 Oct 23;16:137. doi: 10.1186/s12933-017-0622-7 (PMC5654048; doi:10.1186/s12933-017-0622-7)
Supplement: Supplementary file 4 — Additional file 4: Table S1. Selected outcomes from SGLT2 inhibitor CVOTs. [file 12933_2017_622_MOESM4_ESM.docx]

| **Study** | **EMPA-REG**  **Empagliflozin [30, 33]** | **CANVAS (pooled)**  **Canagliflozin [43]** |
| --- | --- | --- |
| **3P-MACE** | **0.86***  0.74; 0.99 | **0.86***  0.75; 0.97 |
| **CV death** | **0.62***  0.49; 0.77 | 0.87^†^  0.72; 1.06 |
| **Non-fatal MI** | 0.87  0.70; 1.09 | 0.85  0.69; 1.05 |
| **Non-fatal stroke** | 1.24  0.92; 1.67 | 0.90  0.71; 1.15 |
| **Hospitalized HF** | **0.65***  0.50; 0.85 | 0.67  0.52; 0.87 |
| **All cause death** | **0.68***  0.57; 0.82 | 0.87^†^  0.74, 1.01 |
| **New or worsening nephropathy (composite renal outcome)** | **0.61***  0.53; 0.70 | 0.60  0.47; 0.77 |
| **New-onset persistent macroalbuminuria** | **0.62***  0.54; 0.72 | 0.73  0.67; 0.79 |
| **Doubling of serum** **creatinine** | **0.56***  0.39; 0.79 | NR |
| **Initiation of renal replacement** **therapy** | **0.45***  0.21; 0.97 | NR |

Additional Table S1 Selected outcomes from SGLT2 inhibitor CVOTs

NR, not reported
New or worsening nephropathy defined as:
**EMPA-REG OUTCOME:** progression to macroalbuminuria, doubling of the serum creatinine level, initiation of renal-replacement therapy, or death from renal disease
**CANVAS trial:** 40% reduction in eGFR, renal-replacement therapy, or renal death
Doubling of serum creatinine defined as:
**EMPA-REG OUTCOME trial:** Doubling of serum creatinine level accompanied by eGFR of 45 ml/min/1.73 m2
